# Validity and Reliability of the Semmes-Weinstein Monofilament Test and the Thumb Localizing Test in Patients With Stroke

**DOI:** 10.3389/fneur.2020.625917

**Published:** 2021-01-27

**Authors:** Mabu Suda, Michiyuki Kawakami, Kohei Okuyama, Ryota Ishii, Osamu Oshima, Nanako Hijikata, Takuya Nakamura, Asako Oka, Kunitsugu Kondo, Meigen Liu

**Affiliations:** ^1^Department of Rehabilitation Medicine, Keio University School of Medicine, Tokyo, Japan; ^2^Department of Rehabilitation Medicine, Tokyo Bay Rehabilitation Hospital, Narashino, Japan; ^3^Biostatistics Unit, Clinical and Translational Research Center, Keio University Hospital, Tokyo, Japan

**Keywords:** assessment, rehabilitation, somatosensory disorders, stroke, thumb localizing test, Semmes-Weinstein monofilament test

## Abstract

**Background:** Somatosensory impairment is common in patients who have had a stroke and can affect their motor function and activities of daily living (ADL). Therefore, detecting and treating somatosensory impairments properly is considered to be very important, and various examinations have been developed. However, the reliability and validity of few of them have been verified due to differences in the procedure of each examiner or poor quantification by the examination itself.

**Objective:** We hypothesized that, with fixed procedures two convenient clinical examinations, the Semmes-Weinstein Monofilament Test (SWMT) and the Thumb Localizing Test (TLT), could provide reliable assessments of light touch sensation and proprioception. The purpose of this study was to verify the reliability and validity of these two examinations as indices of somatosensory impairment of the upper extremity (UE) in patients with chronic post-stroke hemiparesis.

**Methods:** Fifty patients with chronic stroke (median time after onset of stroke, 848 [474–1708] days, mean age 57 [standard deviation 14] years) were enrolled at Keio University Hospital from 2017 to 2018. Examiners learned the original method of the SWMT and the TLT rigorously and shared it with each other. The TLT procedure was partially modified by dividing the location of the patient's thumb into four spaces. Two examiners evaluated the SWMT and the TLT for 2 days, and intra-rater and inter-rater reliabilities were calculated using weighted kappa statistics. In addition to this, the evaluator size score of the SWMT was assessed with Bland-Altman analysis to evaluate systematic bias. The Stroke Impairment Assessment Set (SIAS) sensory items were used to assess validity, and Spearman's rank correlation coefficients were calculated.

**Results:** Intra/inter-rater agreements of the SWMT grade score were 0.89 (thumb, 95%CI: 0.83–0.95)/ 0.75 (0.60–0.91) and 0.80 (index finger, 0.67–0.93)/0.79 (0.66–0.92), and of the TLT they were 0.83 (navel level proximal space, 0.71–0.95)/ 0.83 (0.73–0.92), 0.90 (navel level distal space, 0.85–0.96)/ 0.80 (0.69–0.90), 0.80 (shoulder level proximal space, 0.68–0.92)/ 0.77 (0.65–0.89), and 0.87 (shoulder level distal space, 0.80–0.93)/ 0.80 (0.68–0.92) (*P* < 0.001, each item). All of them showed substantial agreement, but the MDC of the SWMT evaluator size was 1.28 to 1.79 in the inter-rater test and 1.94–2.06 in the intra-rater test. The SWMT grade score showed a strong correlation with the SIAS light touch sensation item (*r* = 0.65, *p* < 0.001), as did the TLT with the SIAS position sense item (*r* = −0.70–0.62, *p* < 0.001 each space).

**Conclusions:** The reliability and validity of the SWMT and the TLT were verified. These tests can be used as reliable sensory examinations of the UE in patients with chronic stroke, and especially for the SWMT, it is more reliable for screening.

## Introduction

Somatosensory impairments, such as of touch, temperature, pain, and proprioception, are common in patients who have had a stroke (1, 2). It has been reported that 85% of patients with chronic stroke have impairment of some sensory modality (3), but the observed prevalence varies between studies (4). Somatosensory impairment correlates with motor function and disturbs the control of fine and coordinated upper extremity (UE) movements (5–7) and goal-directed use of the arm (8). This impairment has an effect on the ability to function in activities of daily living (ADL) (9–11) and participation in life activities (12). Furthermore, the longitudinal process of somatosensory recovery has recently been reported (13, 14), and detecting somatosensory impairment is important clinically.

Various examinations have been developed to detect somatosensory impairment. Traditional clinical examinations such as the light touch test (15), up-down test (16), positional mimicry (15), finger finding (15), and so on can be conveniently performed. However, it has been reported that up to 52% of patients had false-negative results with such traditional clinical examinations compared to the non-affected hand (17). Clinical examinations are routinely performed, but they are sometimes inaccurate and insufficient (18). This unreliability might be caused by differences in the procedure among examiners and by poor quantification of the examination itself (15). Therefore, a series of examinations has been invented. The reliability of examinations with special instruments like the Tactile object identification test (19), the Shape/Texture Identification test (20), the Tactile Discrimination Test (17), and the Wrist Position Sense Test (17) has been reported, but they are not usually available in the hospital. The Fugl-Meyer Assessment set (FMA) (21), the Revised Nottingham Sensory Assessment (NSA) (22), the Erasmus-modified NSA (Em-NSA) (23), the Rivermead assessment of somatosensory performance (RASP) (24), and Quantitative sensory testing (QST) (25) were established assessment sets that contained somatosensory evaluations, and their reliability has also been reported (26). However, Lin reported poor to moderate inter-rater reliability of FMA sensory items of the UE (27). The revised-NSA has many items and requires time to evaluate, and the FMA, Em-NSA, and RASP have only three scoring levels, so they cannot describe the deficits in detail. The light touch item of the QST is measured with a Modified von Frey filament, and it can classify the degree of the deficit more, but its reliability in patients with stroke has not yet been verified.

According to a cross-sectional study (28), 93% of 172 occupational therapists and physiotherapists routinely assess sensory impairment in patients with stroke, and another reported that 87–100% of doctors and therapists perform some clinical examinations to evaluate light touch sensation and proprioception (29). However, there have been few studies in which the researchers rigorously followed original methods and evaluated the psychometric properties of clinical examinations of somatosensory function in patients with stroke. We thought that it would be useful to examine these sensations with convenient clinical examinations with fixed procedures, to share them among examiners, and to evaluate their reliability and validity.

In this study, the Semmes-Weinstein Monofilament Test (SWMT) (30) was used to examine light touch sensation of the UE, and the Thumb Localizing Test (TLT) (31) was used for proprioception. The SWMT is considered a simple and inexpensive touch threshold test and is widely used by clinicians to evaluate sensory disturbances of neuropathic diseases, such as diabetes mellitus and carpal tunnel syndrome. Its reliability and validity in patients with those diseases have been confirmed in previous research (32, 33), but in patients with stroke, those of the SWMT as an index of light touch sensation are poorly documented. However, it is easy to quantify sensory disturbances in detail with the SWMT, so it has often been used as a follow-up index for patients with stroke in the latest studies (34, 35).

To evaluate proprioception, we chose the TLT because Hirayama et al. reported that the TLT showed a greater frequency of abnormalities than other physical examinations for proprioception (36), and the TLT deficits were strongly correlated with the deficits found in joint position and movement (JPM) and the tactile cutaneous localization test (31). However, very few studies have investigated the reliability and validity of the TLT in patients with chronic stroke.

We hypothesized that, with fixed procedures, two convenient clinical examinations, the SWMT and the TLT, could be used to examine light touch sensation and proprioception reliably. The purpose of this study was to verify the reliability and validity of the SWMT and the TLT as indices of somatosensory impairment of the UE in patients with chronic post-stroke hemiparesis.

## Materials and Methods

This research was approved by the Keio University Hospital ethics committee (Approval number 20170123). All participants gave written, informed consent before participation. Before the assessment, they signed the informed consent form, and all their questions about the study were clarified.

### Participants

This research was conducted in parallel with previous randomized, controlled trials or clinical trials (37–40) of rehabilitation of the paretic UE. In this study, all persons who had participated in these trials at Keio University Hospital, Tokyo, Japan, were recruited consecutively from September 2017 to October 2018 until 50 participants were recruited. All participants aged 12–80 years with post-stroke hemiparesis at least 6 months prior to enrollment and who could walk independently without physical assistance in daily life were eligible to participate in the study, as long as their Mini Mental State Examination score was over 20. Exclusion criteria included severe pain or contractures in the paretic UE or cognitive deficits precluding giving informed consent. Patients with visuospatial neglect according to the tape bisection test were excluded. Moreover, patients with suspected deficits of active kinesthesia in the non-affected UE were excluded. The outlines of the studies, including detailed inclusion/exclusion criteria, are presented in the respective reports (37–40). One of the authors extracted the demographic data from the medical records of the participants.

### Procedures

Participants were invited to 2-day measurement sessions at the Rehabilitation Medicine outpatient clinic of Keio University Hospital. The study protocol consisted of three parts: validity, intra-rater reliability, and inter-rater reliability.

The first part investigated the validity of the SWMT and the TLT. One physical medicine and rehabilitation physician performed the SWMT, TLT, and the Stroke Impairment Assessment Set (SIAS) on the 1st day. The SIAS is a standardized measure of stroke impairment consisting of 22 subcategories. Inter-rater reliability of SIAS sensory items has been reported (41), and they were used as the external criteria for the examination of validity (42). As to whether these two tests and SIAS sensory items could measure the same modality, it is very difficult to measure exactly the same modality in the sensory test. SIAS sensory items were adopted because they were different tests, but there seem to be overlapped modalities with the SWMT and TLT.

The second part was intra-rater reliability. Examiner A performed the SWMT and TLT twice, with each examination performed within an interval of 2 days.

The third part was inter-rater reliability. Two physical medicine and rehabilitation physicians (A and B) performed the SWMT and TLT. Examiner B performed them the same day as the first or second examination of Examiner A. The raters were blinded to each other's results. The time period of the examinations was not fixed in any part, but the interval between the examinations by A and B was to be within 8 h in the third part.

On the 1st day, the examiner who saw the participant first examined the modified Ashworth scale (MAS) (43) and the FMA-UE (21) in addition to the sensory tests. The modified Ashworth scale was used as a measure of resistance to passive movement, and the FMA-UE was used as a measure of the severity of motor impairment of the paretic UE.

### Instruments

A manual of procedures was developed, as below, to ensure that the examiners did not perform the tests in their own ways. The manual was referred to as the original method. Then, the study personnel were trained. Examiners performed every test with each other adhering to the manual and confirming every procedure. Afterwards, each examiner then performed them with the chief of research and was corrected until the method was properly performed.

### SWMT

The Touch-Test Sensory Evaluator (North Coast Medical, Inc., Morgan Hill, CA, USA) was used to measure the level of light touch sensation of the tips of the thumb and index finger. This SWMT kit consists of 20 flexible nylon monofilaments of constant length, but varying in diameter. They are labeled so as to give a linear scale of perceived intensity (1.65–6.65) using a logarithmic scale of applied force: labeled number = Log10 of (10 × force in milligrams). The more the labeled number increases, the thicker the filament becomes, and the more pressure is necessary to bend the filament. The filaments are classified from grade 1 to 5 according to their thickness: 1.65–2.83 = grade 5, 3.22–3.61 = grade 4, 3.84–4.31 = grade 3, 4.56–6.45 = grade 2, and 6.65 = grade 1 (Table 1). These numbers and evaluations are cited from “Touch-Test Sensory Evaluator Instructions,” 2011 North Coast Medical, Inc., Morgan Hill, CA, USA.

**Table 1:** Description of the filaments of the Semmes-Weinstein Monofilament Test.

**Evaluator size**	**Target force in grams**	**Thresholds**	**Grade**
1.65	0.008	Normal	5
2.36	0.02		
2.44	0.04		
2.83	0.07		
3.22	0.16	Diminished Light Touch	4
3.61	0.4		
3.84	0.6	Diminished Protective Sensation	3
4.08	1		
4.17	1.4		
4.31	2		
4.56	4	Loss of Protective Sensation	2
4.74	6		
4.93	8		
5.07	10		
5.18	15		
5.46	26		
5.88	60		
6.10	100		
6.45	180		
6.65	300	Deep Pressure Sensation only	1

*Evaluator size, gram force (needed to bend them), the clinical meaning of each filament, and the grade (1–5) used for statistical analyses. These numbers and evaluations are cited from ‘Touch-Test Sensory Evaluator Instructions' 2011 North Coast Medical, Inc., Morgan Hill, CA, USA*.

In a quiet consultation room, the patient was seated at the opposite side of the table from the examiner. The patient's affected arm was supinated and rested on the padded surface table at the level of the navel, and the patient's vision was occluded by a curtain. The testing procedure was explained to the patient, who was then instructed to close his or her eyes and respond when the patient felt being touched by saying “yes” or raising the non-affected hand. The examiner pressed each filament slowly at a perpendicular angle against the skin of the thumb and the index finger until it bowed, held it in place for 1.5 s, and then removed it slowly. For filaments from 1.65 to 4.08, the examiner applied this procedure in the same location up to three times to elicit a response, and for filaments 4.17 through 6.65, they were each applied once only. The examiner decided that it had been sensed by the patient by a single correct timing response with any filament. The interval of pressing filaments was randomized. The examiner began the procedure with the 2.83 filament. [The monofilament number 2.83 was defined as the cut-off for normal sensation (44)]. If the 2.83 filament was felt, lighter filaments were applied in sequence until one was not felt. If the 2.83 filament was not felt, thicker filaments were applied in the same way until one was felt. Finally, the examiner recorded the evaluator scale and grade of the lightest filament that was felt by the patient.

### TLT

**Figure 1:**
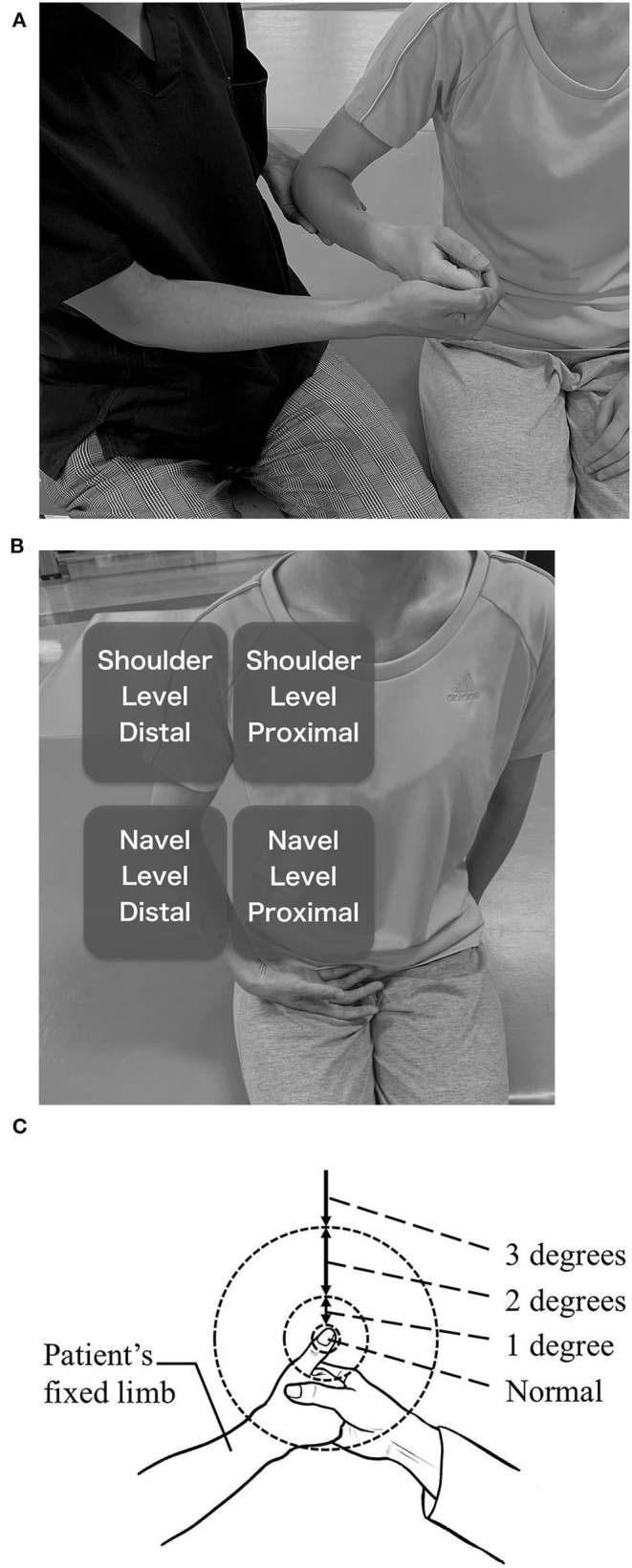
Thumb Localizing Test (TLT). **(A)** positioning of the paretic UE by the examiner. **(B)** four spaces in which the paretic UE is placed. Distal spaces are not far from the trunk because the reaching limb should be able to reach them without difficulty. **(C)** rating of positive results. 1 degree, Once the thumb and index finger of the reaching limb reached several centimeters away from the tip of the thumb of the fixed limb then found themselves not reached to the tip. After that, they make a course correction and finally reach the tip. It is also rated 1 degree if the course of the reaching limb was not linear though the reaching limb could reach to the tip at one time. 2 degrees, The thumb and index finger of the reaching limb reach a place more than a several centimeters from the tip of the thumb of the fixed limb, and move in the air searching for the thumb. Or they accidentally hit the thumb or other fingers of the fixed limb and reach the tip of the thumb tracing over the skin. It is also the case that the patient succeeds in the procedure by moving the thumb of the fixed limb even though he or she was instructed not to do that. 3 degrees, The reaching limb hits the forearm of the fixed limb and reaches the thumb tracing over the skin or the reaching limb moves in the air without finding the thumb and finally the patient abandons the test.

The TLT manual was established following previous reports (31, 45). Prior to the main test, a pretest was performed to confirm that the TLT could be done correctly. The pretest was performed subsequent to the SWMT. The examiner gripped the paretic hand of the patient and made it into a fist, but with the patient's thumb outside the fist. Then, the examiner held the patient's elbow with the examiner's other hand that was not gripping the patient's fist and fixed the patient's paretic UE (fixed limb) to any position (Figure 1A). Afterwards, the examiner asked the patient to relax the fixed limb and pinch the tip of the thumb of the fixed limb with the opposite thumb and index finger (reaching limb). The position of the fixed limb was set to the range where the hand of the reaching limb could reach it without difficulty. The examiner did this pretest with the patient's eyes open in order to check the patient's understanding and confirm no motor paresis, ataxia, or involuntary movements of the reaching limb.

After the pretest was successfully completed, the main test was performed. The fixed limb was moved enough for a few seconds passively and randomly positioned to one of four spaces (described later). Then, the thumb was searched by the reaching limb in the same way as in the pretest, but the eyes of the patient were closed throughout the main test. Although the position of the fixed limb was not described in the original method, the range where the hand of the reaching limb could reach without difficulty was divided into four spaces, and the fixed limb was placed into one of those spaces in this study. The four spaces were the dorsal and distal spaces at the patient's navel level and at the shoulder level (Figure 1B). The examiner performed this procedure several times to complete more than three tests in each space.

When the reaching limb could reach the fixed limb rapidly and linearly, the result was considered negative. Positive results were rated from 1 to 3 as follows by the level of average disability of several procedures (Figure 1C).

1 degree = Once the thumb and index finger of the reaching limb reached several centimeters away from the tip of the thumb of the fixed limb and then patients found themselves having not reached the tip. After that, they make a course correction and finally reach the tip. It is also rated 1 degree if the course of the reaching limb was not linear, though the reaching limb could reach the tip in a single attempt. (“Several centimeters away” was defined as “within the length of the thumb” in this study.)

2 degrees = The thumb and index finger of the reaching limb reach a place more than several centimeters from the tip of the thumb of the fixed limb, and move in the air searching for the thumb, or patients accidentally hit the thumb or other fingers of the fixed limb and reach the tip of the thumb tracing over the skin. It is also the case that the patient succeeds in the procedure by moving the thumb of the fixed limb even though he or she was instructed not to do so.

3 degrees = The reaching limb hits the forearm of the fixed limb and reaches the thumb tracing over the skin, or the reaching limb moves in the air without finding the thumb, and, finally, the patient abandons the test.

After several attempts to search for the thumb, the result was determined as the median value of the scores in each space. The results are reported for all spaces.

### Stroke Impairment Assessment Set (SIAS)

The SIAS is a standardized measure of stroke impairment consisting of 22 subcategories. The sensory items were selected. The SIAS sensory items can evaluate light touch sensation and position sense of the affected UE. Light touch sensation is checked on the palm of the hand rubbed by the finger of the examiner. The examiner evaluates how strongly the patient senses compared to the unaffected hand and rates it from 0 to 3, with 0 indicating anesthesia, one severe or moderately disturbed, two slightly disturbed or paresthesia, and three normal.

Position sense is evaluated with the affected thumb (originally the index finger or thumb). The examiner uses his or her hand to pinch the side tip of the affected thumb of the patient and moves his or her hand up or down slowly. The patient then identifies the direction: up or down. This procedure is performed with the patient's eyes closed. Position sense is rated 0–3, as below (46).

0 = No position change is detected by the patient even with a full range of passive motion.

1 = The patient recognizes the direction of movement with a full range of passive motion.

2 = The patient recognizes the direction of movement with a motion >10% of the full range of motion.

3 = The patient recognizes the direction of movement even with a motion <10% of the full range of motion.

### Data Analyses

Each score of the SWMT was classified according to the aforementioned grades (Table 1) and analyzed. The data of Examiner A's day 1 were used as the baseline characteristics.

The differences between items of the SWMT grade (thumb vs. index) and the TLT score (four spaces) were assessed with the Wilcoxon signed-rank test and the Friedman test.

Intra-rater and inter-rater relative reliabilities of the TLT score and the SWMT grade were assessed by weighted kappa statistics. A kappa value >0.8 indicates excellent agreement, 0.6/0.8 indicates good agreement, 0.4/0.6 indicates moderate agreement, and <0.4 indicates poor agreement (47).

Absolute reliability of the evaluator size of the SWMT was assessed using Bland-Altman analysis to confirm whether systematic bias (i.e., fixed bias and proportional bias) was present. (The SWMT grade and TLT were not assessed because their ratings use ordinal scales.) If the 95% confidence intervals (CI) of the differences between two tests included zero, it was considered that there was no fixed bias. If the coefficient derived by linear regression of the difference and mean was equal to zero (i.e., the *p*-value was >0.05), it was considered that there was no proportional bias. The limits of agreement were derived as follows:

$$\begin{array}{l} \bar{d}\;\pm \;t\;\times \;S_{diff}\;\times \;\sqrt{1+1/n} \end{array}$$

where $\bar{d}$ is the mean difference, *t* is the value of Student's *t* statistic corresponding to a two-sided *p* = 0.05 at *n*-1 degrees of freedom, *S*_*diff*_ is the standard deviation of the difference, and *n* is the sample size. This formula is suitable for the present sample size (48). The measurement errors were quantified by the standard error of measurement (SEM), which was derived by dividing the standard deviation of the mean differences by $\sqrt{2}$. The minimal detectable change (MDC), which indicates the magnitude of change necessary to exceed the measurement error, was derived by SEM × 1.96 $\times \sqrt{2}$.

The validity of the TLT score and that of the SWMT grade were assessed with Spearman's rank correlation coefficient for the relationship between the scores and SIAS sensory items. The 95% CI was calculated based on Fisher's transformation. The strength of correlation is considered very strong if the coefficient ranges from 0.8 to 1, strong from 0.5 to 0.8, weak from 0.2 to 0.5, and very weak when <0.2 (49).

IBM SPSS Statistics for MAC, Version 24.0 (IBM Corp., Armonk, NY, USA) was used for the analyses.

## Results

Fifty patients with chronic stroke (57 ± 8.3 years, range: [20–79] years, 34 male, 16 female, affected hemisphere: 21 right, 27 left, 2 both) participated (characteristics listed in Table 2). The median time after stroke onset was 848 days (IQ range: [474–1708] days). Of the participants, 26 suffered from hemorrhage, 21 from infarction, two from subarachnoid hemorrhage, and one from tumor. The median MAS value of the elbow and wrist was two (IQ range: [2–3]), and that of the finger was 2.5 (IQ range: [1–3]). (For the MAS, Score 1+ was transformed to 2, and scores 2 and 3 were transformed to 3 and 4 in this calculation.) The median SIAS light touch and position sense scores were 2 (IQ range: [1.25–3]) and 3 (IQ range: [1–3]), respectively. The mean total FMA-UE score was 30.5 (SD = 13.5, range: [9–63]).

**Table 2:** Participants' stroke characteristics.

Age, year	57 (14, 20–79)^a^
Time after stroke onset, day	848 [474–1708]^b^
Gender	Male 34 (68)^c^ Female16 (32)^c^
Stroke characteristics	
Affected hemisphere	Right 21 (42)^c^
	Left27 (54)^c^
	Both2 (4)^c^
Stroke type	Hemorrhage 26 (52)^c^
	Infarction 21 (42)^c^
	SH 2 (4)^c^
	Tumor 1 (2)^c^
FMA-UE (33 items)	30.5 (13.5, 9–63)^a^
MAS Elbow	2, *n* = 5; 1+, *n* = 16;
	1, *n* = 18; 0, *n* = 11
Wrist	2, *n* = 5; 1+, *n* = 19;
	1, *n* = 15; 0, *n* = 11
Finger	2, *n* = 5; 1+, *n* = 20;
	1, *n* = 9; 0, *n* = 16
SIAS light touch score	3, *n* = 21; 2, *n* = 16;
	1, *n* = 13; 0, *n* = 0
position sense score	3, *n* = 30; 2, *n* = 6;
	1, *n* = 9; 0, *n* = 5
SWMT thumb grade	5, *n* = 20; 4, *n* = 12;
	3, *n* = 7; 2, *n* = 6; 1, *n* = 5
index finger grade	5, *n* = 21; 4, *n* = 11;
	3, *n* = 4; 2, *n* = 10; 1, *n* = 4
TLT navel level, proximal	3, *n* = 7; 2, *n* = 12; 1, *n* = 16; normal, *n* = 15
navel level, distal	3, *n* = 14; 2, *n* = 13;
	1, *n* = 12; normal, *n* = 11
shoulder level, proximal	3, *n* = 7; 2, *n* = 13; 1, *n* = 16; normal, *n* = 14
shoulder level, distal	3, *n* = 16; 2, *n* = 14;
	1, *n* = 14; normal, *n* = 8

SH, subarachnoid hemorrhage; FMA, Fugl-Meyer Assessment; MAS, modified Ashworth scale; SIAS, Stroke Impairment Assessment Set; SWMT, Semmes-Weinstein Monofilament Test; TLT, Thumb Localizing Test.

a mean(SD, range),

b median[interquartile range],

c *n (%)*.

### SWMT

The difference in the SWMT grade between the thumb and index fingers was not significant (Wilcoxon signed-rank test: *P* = 1.00). The inter-rater and intra-rater reliabilities of the SWMT grade are shown in Table 3. The weighted kappa values for inter-rater reliabilities of the SWMT grade were 0.75 (thumb, 95%CI: 0.60–0.91) and 0.79 (index finger, 0.66–0.92), and for intra-rater reliability they were 0.89 (thumb, 0.83–0.95) and 0.80 (index finger, 0.67–0.93) (*P* < 0.001, each item).

**Table 3:** Inter- and intra-rater reliabilities and validities of the Semmes-Weinstein Monofilament Test and the Thumb Localizing Test, and the differences among the four spaces of the Thumb Localizing Test.

	**Inter-rater reliability K (95% CI)**	***p***	**Intra-rater reliability K (95% CI)**	***p***	**Validity V.S. SIAS light touch score R (95% CI)**	***p***	**Validity V.S. SIAS position sense score R (95% CI)**	***p***
SWMTthumb	0.75 (0.60–0.91)	<0.001	0.89 (0.83–0.95)	<0.001	0.57 (0.35–0.73)	<0.001	0.57 (0.34–0.73)	<0.001
SWMTindex finger	0.79 (0.66–0.92)	<0.001	0.80 (0.67–0.93)	<0.001	0.65 (0.46–0.79)	<0.001	0.59 (0.37–0.74)	<0.001
TLTnavel level, proximal	0.83 (0.73–0.92)	<0.001	0.83 (0.71–0.95)	<0.001	−0.52 (−0.70 – –0.28)	<0.001	−0.62 (−0.76 – –0.41)	<0.001
TLTnavel level, distal	0.80 (0.69–0.90)	<0.001	0.90 (0.85–0.96)	<0.001	−0.61 (−0.76 – –0.40)	<0.001	−0.66 (−0.79 – –0.46)	<0.001
TLTshoulder level, proximal	0.77 (0.65–0.89)	<0.001	0.80 (0.68–0.92)	<0.001	−0.54 (−0.71 – –0.30)	<0.001	−0.70 (−0.82 – –0.52)	<0.001
TLTshoulder level, distal	0.80 (0.68–0.92)	<0.001	0.87 (0.80–0.93)	<0.001	−0.61 (−0.76 – –0.39)	<0.001	−0.66 (−0.79 – –0.46)	<0.001

*SWMT, Semmes-Weinstein Monofilament Test; TLT, Thumb Localizing Test*.

**Figure 2:**
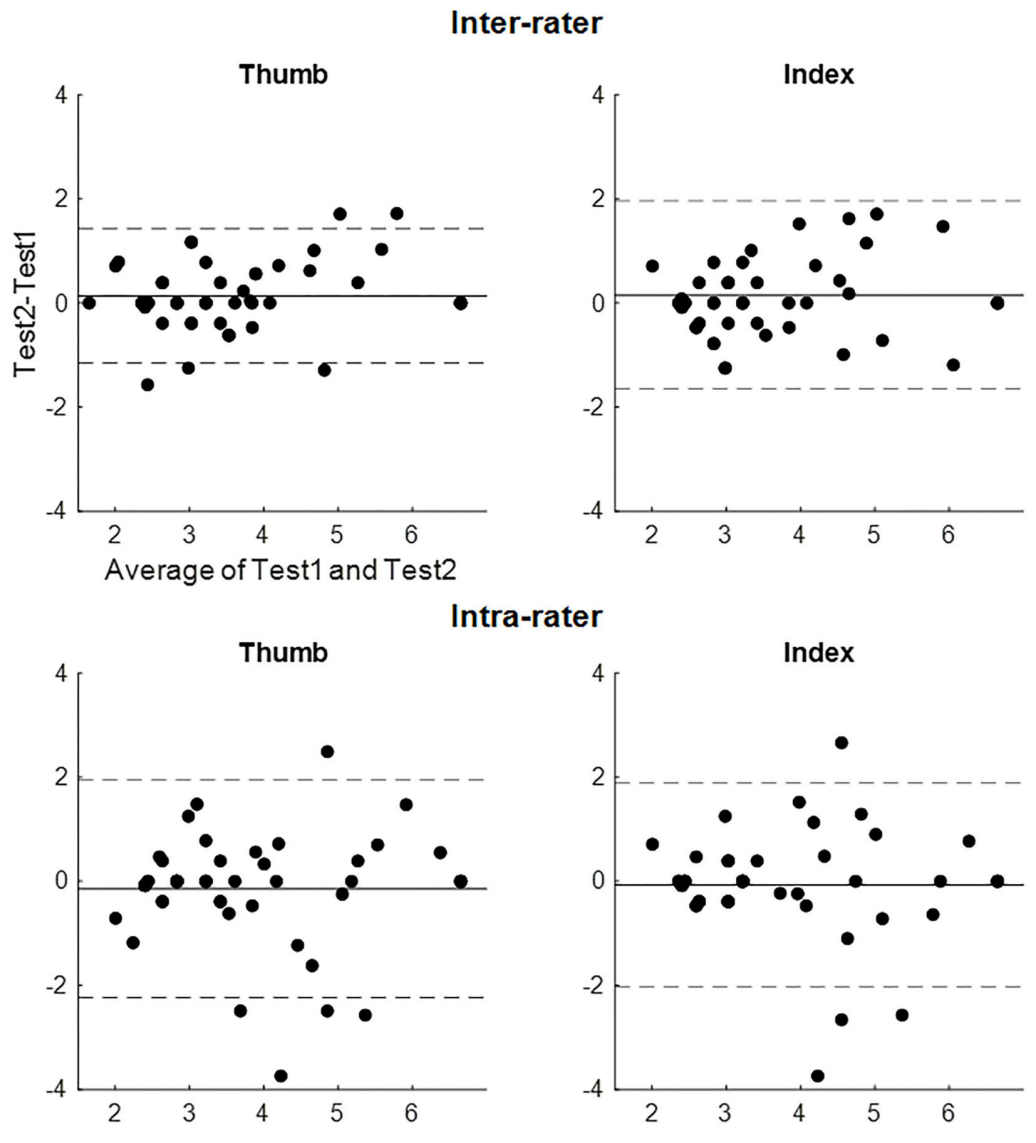
Bland-Altman plot of the evaluator size of the Semmes-Weinstein Monofilament Test. The solid line represents the mean difference. The dashed line represents the upper and lower limits of agreements of the difference.

The absolute reliabilities of the evaluator size of the SWMT (Bland-Altman analysis, SEM, and MDC) are shown in Table 4. Figure 2 shows the Bland-Altman plot of the evaluator size of the SWMT. There was no fixed or proportional bias. The MDC of the evaluator size was 1.28 for the thumb and 1.79 for the index finger in the inter-rater test and 1.94 for the thumb and 2.06 for the index finger in the intra-rater test. With respect to the convergent validity of the SWMT against the SIAS light touch score, Spearman's rho ranged from 0.57 to 0.65 (*p* < 0.001, each item).

**Table 4:** Results for absolute reliability in the evaluator size of the Semmes-Weinstein Monofilament Test.

	**Inter-rater**		**Intra-rater**	
	**Thumb**	**Index**	**Thumb**	**Index**
The 95% CIs of the difference	−0.09–0.37	−0.12–0.43	−0.44–0.15	−0.35–0.21
Linear regression				
β	0.28	0.19	0.18	0.19
*t* value	1.45	0.97	−0.01	−0.43
*p*-value	0.15	0.34	0.99	0.67
The Limits of agreement				
Upper	−1.16	−1.66	−2.23	−2.03
Lower	1.43	1.96	1.94	1.89
SEM	0.46	0.65	0.74	0.70
MDC	1.28	1.79	2.06	1.94

*CIs, confidence intervals; SEM, standard error of measurement; MDC, minimal detectable change*.

### TLT

Differences among the four spaces were significant (Friedman test: *P* < 0.001). The median for each space was two for the proximal spaces and three for the distal spaces. The difference between each space was also significant for both proximal spaces vs. both distal spaces (Wilcoxon signed-rank test: *P* < 0.01), but it was not significant for both navel level spaces vs. both shoulder level spaces (Wilcoxon signed-rank test: *p* = 0.68 and 0.16). The weighted kappa values for inter-rater reliabilities of each space of the TLT were 0.83 (navel level, proximal, 95%CI: 0.73–0.92), 0.80 (navel level, distal, 0.69–0.90), 0.77 (shoulder level, proximal, 0.65–0.89), and 0.80 (shoulder level, distal, 0.68–0.92). For intra-rater reliability, they were 0.83 (navel level, proximal, 0.71–0.95), 0.90 (navel level, distal, 0.85–0.96), 0.80 (shoulder level, proximal, 0.68–0.92), and 0.87 (shoulder level, distal, 0.80–0.93). Each *P*-value was < 0.001 (see Table 3). With respect to the four spaces of the TLT, Spearman's rho ranged from −0.61 to −0.52 with SIAS light touch sensation and from −0.70 to −0.62 with SIAS position sense (*p* < 0.001, each item).

## Discussion

Similar to other examinations for somatosensory impairment, the reliability and validity of the SWMT and the TLT have not been well-established (50). In the present study, the reliability and validity of the SWMT and the TLT for evaluating sensory disturbances of patients with chronic post-stroke hemiparesis were verified.

Both thumb and index fingers were examined with the SWMT grade, and intra/inter-rater agreements were more than good for each finger. In addition, fixed bias and proportional bias of the evaluator size of the SWMT were not present based on the results of Bland-Altman analysis. The MDC of the evaluator size was 1.28–1.79 in the inter-rater test and 1.94–2.06 in the intra-rater test. This implies that, even when the examiners learned the fixed procedures, the result of the evaluator size varied almost ±2 among the examiners. As shown in Table 1, two difference in the evaluator size means at least a one-grade difference, indicating that use of evaluator size may be inadequate for detecting small changes, such as when following-up patients' somatosensory disturbance or treatment response. Recent studies have also used the evaluator size as a follow-up index for light touch sensation in patients with stroke, as mentioned in the introduction, so further modification of the SWMT should be conducted in a future study to detect small changes.

The SWMT grade of both fingers showed a strong correlation with the SIAS light touch item, and there was no significant difference between the thumb and index finger. Both fingers may be used as indices of light touch sensation in clinical practice. In other words, if patients have keratinization on their fingers or they have difficulty in extending their fingers due to spasticity, another finger, the thumb or index finger, can be selected. According to these findings, the SWMT grading is a practical clinical examination as a convenient screening tool.

The TLT was performed in four spaces to investigate whether the scores depended on the position where the fixed limbs were placed. The scores of each space were significantly different, and the distal space showed a larger grade than the proximal space. It was not possible to determine whether the proximal space is likely to give false-negative results or the distal space gives false-positive results, but it is reasonable that accidental hits may increase when the fixed limb is located in the proximal space, because the reaching limb is closer to the fixed limb. Therefore, we suggest that the examiner place the fixed limb in various positions but adopt the scores of the distal space, in addition to the original TLT method. Excellent inter-rater agreement was observed, and good to excellent intra-rater agreement was seen in every space. There was a strong correlation between the score of each space and both of the SIAS sensory items. Furthermore, they correlated more with SIAS position sense than with light touch sensation. Thus, the TLT is considered to be sufficiently practical to evaluate proprioception, but it is possible that the TLT might examine wider modalities of proprioception than other assessments such as the JPM, FMA, Em-NSA, and RASP, because these tests evaluate whether patients can sense the movement of each joint. The TLT was considered to examine the relative positional relationship in space between a passively fixed thumb and the body axis as measured by motor tasks of the contralateral reaching limb (31). Therefore, other tests should be performed for screening, and the TLT should also be examined to avoid overlooking proprioceptive disturbance.

As far as we could find, the “Thumb Localization Test” and “Thumb Localizing Test” were called the TLT. These two tests are completely different. The “Thumb Localization Test” appeared to have been proposed and its reliability and validity examined by Rand et al. (51). This is similar to the traditional finger (thumb) finding that patients have to grip their overall affected thumb with their non-affected hand in contrast to pinching the tip of the affected thumb with the opposite thumb and index finger in the “Thumb Localizing Test.” In the present study, the “Thumb Localizing Test” proposed by Hirayama et al. (31) was used as above; pinching the tip can eliminate accidental hits much more than gripping the whole thumb.

Though somatosensory impairments are regarded as an indicator of a poor prognosis, those of patients with chronic stroke have not been subjects of interest (52). Serrada et al. reported that sensory-based interventions have been overlooked, although they are likely to form a critical component of stroke recovery in post stroke patients (53). They also mentioned that there was some evidence to support the use of passive sensory techniques for improving sensation and sensorimotor function. Regarding the UE of patients with chronic stroke, not much evidence has been reported, but Byl et al. reported that their sensorimotor training improved both sensory and motor functions (54), and Tashiro et al. showed improvement in the TLT and somatosensory evoked potentials, in addition to motor function, after intervention with neuromuscular electrical stimulation (55). The patients can successfully proceed to receiving such rehabilitation if their somatosensory deficits are properly identified. The present findings can contribute to this: the SWMT and the TLT are sufficiently reliable sensory examinations for screening the paretic UE in patients with chronic stroke. We will investigate how to modify these tests as indices also for small changes in a future study.

The present study had several limitations. First, the SIAS sensory items were used for assessing concurrent validity of the SWMT and the TLT. The SIAS is a semi-quantitative assessment set and not a standard method for evaluating sensory disturbance (26). We plan to investigate the correlation between these two tests and more quantitative assessments in our next study. Second, the unbalanced sample was also a limitation. For example, the study sample did not include the most severe cases, such as bed-bound patients and relatively mild cases that did not need rehabilitation. The participants were recruited in a single center for rehabilitation of the UE, and the unbalanced patient characteristics may affect the generalization of the results. Third, only the UE of the patients was investigated, so applying the present findings to the trunk or lower extremity requires further research.

## Data Availability Statement

The raw data supporting the conclusions of this article will be made available by the authors, without undue reservation.

## Ethics Statement

The studies involving human participants were reviewed and approved by Keio University Hospital ethical committee (Approval number 20170123). The patients/participants provided their written informed consent to participate in this study.

## Author Contributions

MS, MK, and KO were chiefly responsible for the concept and design of the study, data analysis and interpretation, and drafting the manuscript. RI contributed to verifying the statisitical methods. OO, NH, TN, and AO contributed to data acquisition and analyses. ML and KK contributed to the concept and design of the study. All authors revised the article for critical content, approved the final version for publication, and agreed with its submission to Frontiers in Neurology for publication.

## Conflict of Interest

The authors declare that the research was conducted in the absence of any commercial or financial relationships that could be construed as a potential conflict of interest.
