# Supplementary material for: Validity and Reliability of the Semmes-Weinstein Monofilament Test and the Thumb Localizing Test in Patients With Stroke
Source: Front Neurol. 2021 Jan 27;11:625917. doi: 10.3389/fneur.2020.625917 (PMC7873561; doi:10.3389/fneur.2020.625917)
Supplement: Supplementary file 1 [file Table_1.DOCX]

The operation manual of the SWMT and the TLT

# The Semmes-Weinstein Monofilament Test

In a quiet consultation room, the patient is seated at the opposite site of the table from the examiner. The patient’s affected arm is supinated and rested on the padded surface table at the level of the navel, and the patient’s vision is occluded by a curtain. The testing procedure is explained to the patient, who is then instructed to close his or her eyes and respond when the patient felt being touched by saying “yes” or raising the non-affected hand.

The examiner presses each filament slowly at a perpendicular angle against the skin of the thumb and the index finger until it bows, holds it there for 1.5 seconds, and then removes it slowly. For filaments from 1.65 to 4.08, the examiner applies this procedure in the same location up to three times to elicit a response, and for filaments 4.17 through 6.65, they are each applied once only.

The examiner begins the procedure with the 2.83 filament. (The monofilament number 2.83 is defined as the cut-off for normal sensation.) The examiner decides that it has been sensed by the patient by a single correct timing response with any filament. If the 2.83 filament is felt, lighter filaments are applied in sequence until one is not felt. If the 2.83 filament is not felt, thicker filaments are applied in the same way until one is felt. The interval of pressing filaments should be randomized. Finally, the examiner records the evaluator scale and grade (see **Supplementary Table.1**) of the lightest filament that was felt for the patient.

**Supplementary Table 1.** The description of the filaments of Semmes-Weinstein Monofilament Test

| Evaluator size | Target force  in grams | Thresholds | Grade |
| --- | --- | --- | --- |
| 1.65 | 0.008 | Normal | 5 |
| 2.36 | 0.02 |  |  |
| 2.44 | 0.04 |  |  |
| 2.83 | 0.07 |  |  |
| 3.22 | 0.16 | Diminished Light Touch | 4 |
| 3.61 | 0.4 |  |  |
| 3.84 | 0.6 | Diminished Protective Sensation | 3 |
| 4.08 | 1 |  |  |
| 4.17 | 1.4 |  |  |
| 4.31 | 2 |  |  |
| 4.56 | 4 | Loss of Protective Sensation | 2 |
| 4.74 | 6 |  |  |
| 4.93 | 8 |  |  |
| 5.07 | 10 |  |  |
| 5.18 | 15 |  |  |
| 5.46 | 26 |  |  |
| 5.88 | 60 |  |  |
| 6.10 | 100 |  |  |
| 6.45 | 180 |  |  |
| 6.65 | 300 | Deep Pressure Sensation only | 1 |

Evaluator size, gram force (needed to bend them), the clinical meaning of each filament, and the grade (1-5). These numbers and evaluations are cited from ‘Touch-Test Sensory Evaluator Instructions’ © 2011 North Coast Medical, Inc.

# The Thumb Localizing Test

Prior to the main test, a pretest is performed. The pretest confirms that the TLT can be done correctly. The pretest is performed subsequent to the SWMT. The examiner grips the paretic hand of the patient and makes it into a fist, but with the patient’s thumb outside the fist. Then, the examiner holds the patient’s elbow with the examiner’s other hand that is not gripping the patient’s fist and fixes the patient’s paretic UE (fixed limb) to any position (**Supplementary Figure A**). Afterwards, the examiner asks the patient to relax the fixed limb and pinch the tip of the thumb of the fixed limb with the opposite thumb and index finger (reaching limb). The position of the fixed limb is set to the range where the hand of the reaching limb can reach it without difficulty. The examiner does this pretest with the patient’s eyes open in order to check the patient’s understanding and confirm no motor paresis, ataxia, or involuntary movements of the reaching limb.


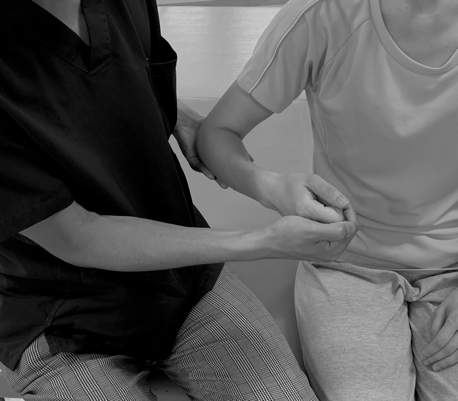


**Supplementary Figure A.** Positioning of the paretic UE by the examiner.

After the pretest was successfully done, the main test is performed. The fixed limb is moved enough for a few seconds passively and randomly positioned in the range where the hand of the reaching limb can reach without difficulty. Then the thumb is searched by the reaching limb in the same way as in the pretest, but the eyes of the patient are closed throughout the main test. The examiner does this procedure several times and he or she should place the fixed limb both proximal and distal space in a series of procedures.

When the reaching limb can reach the fixed limb rapidly and linearly, the result is considered negative. Positive results are rated from 1 to 3 as follows by the level of average disability of several procedures (**Supplementary Figure B**).

1 degree = Once the thumb and index finger of the reaching limb reached several centimeters away from the tip of the thumb of the fixed limb and then patients found themselves having not reached the tip. After that, they make a course correction and finally reach the tip. It is also rated 1 degree if the course of the reaching limb was not linear, though the reaching limb could reach the tip in a single attempt.

2 degrees = The thumb and index finger of the reaching limb reach a place more than a several centimeters from the tip of the thumb of the fixed limb, and move in the air searching for the thumb. Or they accidentally hit the thumb or other fingers of the fixed limb and reach the tip of the thumb tracing over the skin. It is also the case that the patient succeeds in the procedure by moving the thumb of the fixed limb even though he or she was instructed not to do that.

3 degrees = The reaching limb hits the forearm of the fixed limb and reaches the thumb tracing over the skin or the reaching limb moves in the air without finding the thumb and finally the patient abandons the test.

After several times of searching for the thumb, the result is determined as the median value of the scores. (We recommend only the scores of distal space should be adopted and calculated.)


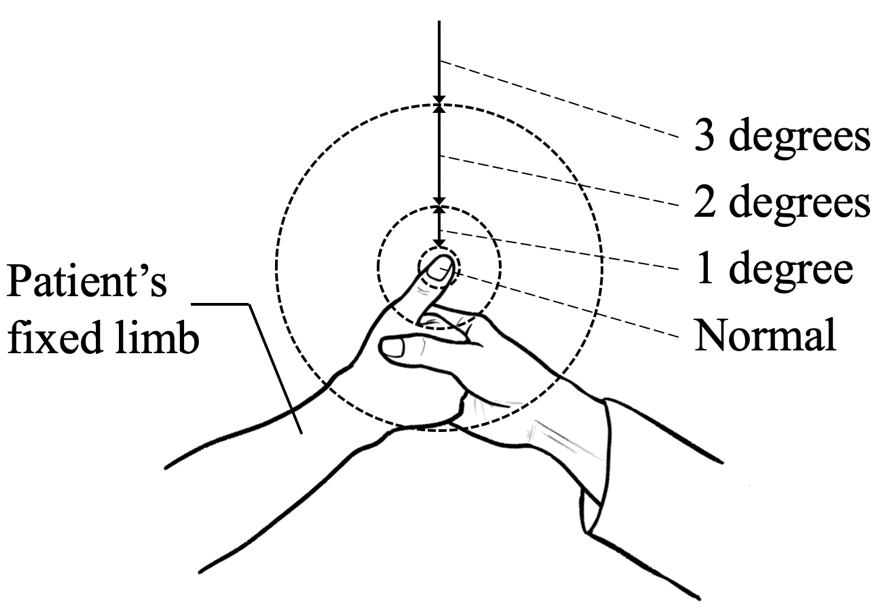


**Supplementary Figure B.** The rating of the TLT.
